# Detection of African swine fever virus antibodies in serum using a pB602L protein-based indirect ELISA

**DOI:** 10.3389/fvets.2022.971841

**Published:** 2022-09-23

**Authors:** Yang Yang, Qiqi Xia, Qin Sun, Yan Zhang, Yuhao Li, Xiaochun Ma, Zhixin Guan, Junjie Zhang, Zongjie Li, Ke Liu, Beibei Li, Donghua Shao, Yafeng Qiu, Zhiyong Ma, Jianchao Wei

**Affiliations:** ^1^Shanghai Veterinary Research Institute, Chinese Academy of Agricultural Sciences, Shanghai, China; ^2^College of Animal Science, Yangtze University, Jingzhou, China

**Keywords:** African swine fever virus, indirect ELISA, B602L, prokaryotic expression system, antibodies in serum

## Abstract

African Swine Fever (ASF) is an acute, highly contagious and deadly infectious disease that has a huge impact on the swine industry. It is caused by the African swine fever virus (ASFV). The most acute forms of ASF in domestic pigs have mortality rates of up to 100%. The lack of a commercial vaccine and effective therapeutic drugs has brought great challenges to the prevention and control of ASF. Current, the African swine fever virus requires a huge amount of detection, so there is a need for more sensitive and accurate detection technology. The protein pB602L, as a late non-structural protein, has a high corresponding antibody titer and strong antigenicity in infected swine. In this research, the B602L gene was constructed into the pColdI prokaryotic expression vector, and prokaryotic expression of the soluble pB602L protein was induced by IPTG. Western blot analysis demonstrated that the protein had strong immunogenicity. We established an indirect ELISA method for the detection of anti-ASFV using purified recombinant pB602L protein as antigen. The detection method showed excellent specificity without cross-reactions with antibodies against PRRSV, CSFV, JEV, and GETV. The method could detect anti-ASFV in serum samples that were diluted up to 6,400 times, showing high sensitivity. The coefficients of variation of the intra-assay and inter-assay were both <10%. The assays had excellent specificity, sensitivity, and repeatability. In summary, we developed an accurate, rapid, and economical method for the detection of anti-ASFV in pig serum samples with great potential for ASF monitoring and epidemic control.

## Introduction

African swine fever (ASF) is a highly contagious hemorrhagic and transboundary viral disease of swine for which currently no preventive vaccine is available (1). It is a devastating disease with mortality rates close to 100% in young herds, causing severe socioeconomic losses. Since it was first reported in Kenya in 1921 (2), ASF has become endemic in many African countries (3). Recently, ASF emerged in Asian countries, including China (4), where it spread rapidly to many parts of the country (5). At present, ASF continues to place a huge burden on the pig industry in the affected areas (6).

Current approaches to control and prevent ASF include early diagnosis, rapid elimination of infectious sources, and interruption of transmission routes. Therefore, the development of rapid, sensitive, and field-deployable African swine fever virus (ASFV) assays are important for disease surveillance and control (7, 8). Currently, there are several early diagnostic techniques available to detect ASFV, including PCR, loop-mediated isothermal amplification, fluorescence quantitative PCR, colloidal gold rapid strip, hemadsorption, enzyme-linked immunosorbent assay (ELISA), and other serological tests (9). Serological testing is been widely used in ASF eradication and control programs; since no vaccine is available, the presence of ASF antibodies implies previous infection. Therefore, serology is suitable for detecting potential virus-carrying animals in epidemic situations. ELISA is the most commonly used serological test, so it can be widely used for screening purposes (10).

ASFV is the sole member of the genus Asfarvirus within the family Asfarviridae and is a unique and genetically complex virus (11). ASFV is a large, double-stranded, linear DNA virus with a complex structure. The genome size is about 170–190 kb. ASFV encodes more than 150 proteins, of which more than 50 are structural proteins (12). ASFV can be divided into 23 different genotypes according to the ASFV B646L gene encoding the p72 major capsid protein (13). Morphologically, ASFV has a icosahedral shape and a complex structural architecture composed of several concentric layers (14, 15). ASFV infects mononuclear macrophages mainly through megalocytosis and clathrin-mediated endocytosis (1). pB602L is a nonstructural protein that functions as a molecular chaperone of the major structural protein p72, forming aberrant “zipper-like” structures instead of icosahedral virus particles in the absence of pB602L (16). B602L contains a central variable region (CVR) that allows frequently subgenotyping of ASFV isolates based on this region (17). The central variable region (CVR) within the B602L gene of the African swine fever virus (ASFV) is highly polymorphic within the ASFV genotypes defined by sequencing of the C-terminal end of the p72 locus (17, 18). Previous studies have shown that pB602L is strongly antigenic and can be used to develop diagnostic tools for ASFV. Previously, a pB602L-based ELISA assay was employed to detect serum antibodies against ASFV, and the test results were mostly consistent with those obtained using the gold standard, western blot assays (16). B Gutiérrez-Castañeda et al. showed that pB602L is recognized by hyperimmune antisera from domestic pigs and bushpigs at late time points after infection, suggesting it may be useful diagnostically to distinguish animals persistently infected with virus (17). pB602L may be a suitable target for the development of diagnostic tools to assess the humoral immune responses to these vaccines, as antibodies against pB602L are only produced after this protein is expressed in host cells (1, 17).

In this study, we successfully expressed and purified the ASFV pB602L protein and established an indirect ELISA method for the detection of anti-ASFV using purified pB602L as a diagnostic antigen, laying the foundation for the development of early diagnosis kits for ASFV and functional research of the pB602L protein.

## Materials and methods

### Serum samples

ASFV (SY-18) infection inactivated sera (*n* = 30) were obtained from the China Animal Health & Epidemiology Center (Qingdao, China). All positive sera were tested by gold standard. And all positive sera were tested by the Svanova ASFV p30 antibody detection kit and the Ingenasa ASFV p72 ELISA kit.

Positive sera against pathogenic Japanese encephalitis virus (JEV), classical swine fever virus (CSFV), Getah virus (GETV), Porcine circovirus 2 (PCV2), Porcine parvovirus (PPV)-, and Pseudorabies virus (PRV) were prepared in our laboratory.

All 120 negative serum samples were collected before the outbreak of ASFV in China from 2016 to 2017 and were confirmed to be negative by the Svanova ASFV p30 antibody detection kit and the Ingenasa ASFV p72 ELISA kit.

### Cloning and expression of pB602L

The transmembrane region of pB602L protein was predicted through TMHMM-2.0 (Denmark, http://www.cbs.dtu.dk/services/TMHMM/). The full-length B602L coding region of ASFV SY-18 (GenBank accession, MH766894.1) was synthesized by Tsingke Biotechnology (Shanghai, China) and amplified using primers. F (5′-CTCGGTACCCTCGAGGGATCCATGGCAGAATTTAATATTGA-3′) and R (5′-GACTGCAGGTCGACAAGCTTCTACAATTCTGCTTTTGTAT-3′).

The recombinant expression plasmid was constructed by ligating the PCR product of the B602L gene into the vector pCold I. The ligation product was transformed into *E.coli* DH5α, and the recombinant plasmid was successfully constructed by sequencing. Next, the constructed plasmids were transformed into *E.coli* BL21, and then protein expression was induced for 14–16 h with 0.5 mM IPTG at 16°C. The recombinant pB602L protein was purified by Ni^+^ affinity chromatography and identified by SDS-PAGE and western blots. ASFV-positive pig serum (dilution of 1:5,000) and anti-His (dilution of 1:5,000) were used as the primary antibodies for the western blot assays.

### Establishment of an indirect ELISA antibody detection method for ASFV pB602L protein

#### Square titration

The working concentration of pB602L protein antigen and serum dilution were optimized using square titration. In short, the antigen pB602L was coated on plates at concentrations of 0.625, 0.32, 0.16, 0.08, and 0.04 μg/mL. Anti-ASFV positive and negative standard sera were diluted at 1:50, 1:100, 1:200, and 1:400. The condition with the highest OD450 ratio of positive and negative sera (P/N value) and an OD450 value of positive sera close to 1.0 was selected as the best working condition.

#### Working conditions for pB602L-ELISA

In addition to optimal pB602L protein coating concentration and serum dilution, different blocking solutions (5% skim milk, 1% gelatin, and 2% BSA) and reaction times of various materials were also explored, and the optimal reaction time of serum was also tested. The optimal concentration and reaction time of HRP-conjugated anti-pig antibody were tested using the following dilutions: 1:2,500, 1:5,000 and 1:10,000. Furthermore, the TMB response time was optimized. The OD450 and P/N values of these groups were compared to determine the best working conditions.

#### Cut-off value for pB602L-ELISA

The optimal cutoff value for iELISA was determined using a total of 64 negative serum samples from ASFV-uninfected swnie. The OD450 values were measured, and statistical analysis was performed to calculate the average value (AV) and standard deviation (SD) of the OD450 values. The cut-off value was determined as AV + 3SD (19, 20).

#### Specificity and sensitivity of pB602L-ELISA

The constructed pB602L-ELISA method was applied against porcine reproductive and respiratory syndrome virus (PRRSV)-, Japanese encephalitis virus (JEV)-, classical swine fever virus (CSFV)-, Getah virus (GETV)-, Porcine circovirus 2 (PCV2)-, Porcine parvovirus (PPV)-, and Pseudorabies virus (PRV)-positive sera, ASFV-positive and -negative sera. We diluted ASFV-positive serum from 1:100 to 1:204,800 to determine the highest dilution of serum. We diluted ASFV-positive serum from 1:100 to 1:204,800 to determine the highest dilution of serum. Based on the critical value, the sensitivity of the constructed ELISA method was appraised. Statistical analysis Coefficient of Variation.

#### Reproducibility of pB602L-ELISA

Six random serum samples were randomly selected, and the established pB602L-ELISA assay was used to conduct intra-assays repeatability tests on three different ELISA plates coated in the same batch. In addition, three different batches of coated ELISA plates were randomly selected for inter-assay repeatability experiments. Statistical analysis was performed to calculate intra- and inter-assays variation [coefficient of variation (CV)] between runs.

#### Comparison of pB602L-ELISA with commercial kits

All 150 clinical serum samples were tested applying the pB602L-ELISA methed. The results were compared with the results of a commercial ELISA kit for anti-ASFV detection to evaluate the performance of pB602L-ELISA by calculating the concordance rate as follows: [(true positive + true negative) / (true positive + true negative + true negative + false positive) × 100%].

### Statistical analysis

All data were analyzed using Prism 5 software (GraphPad Software, La Jolla, CA, USA). All data were analyzed using a two-tailed Student's *t*-test. *P* < 0.05 was considered statistically significant.

## Results

### Expression and purification of pB602L protein

**Figure 1:**
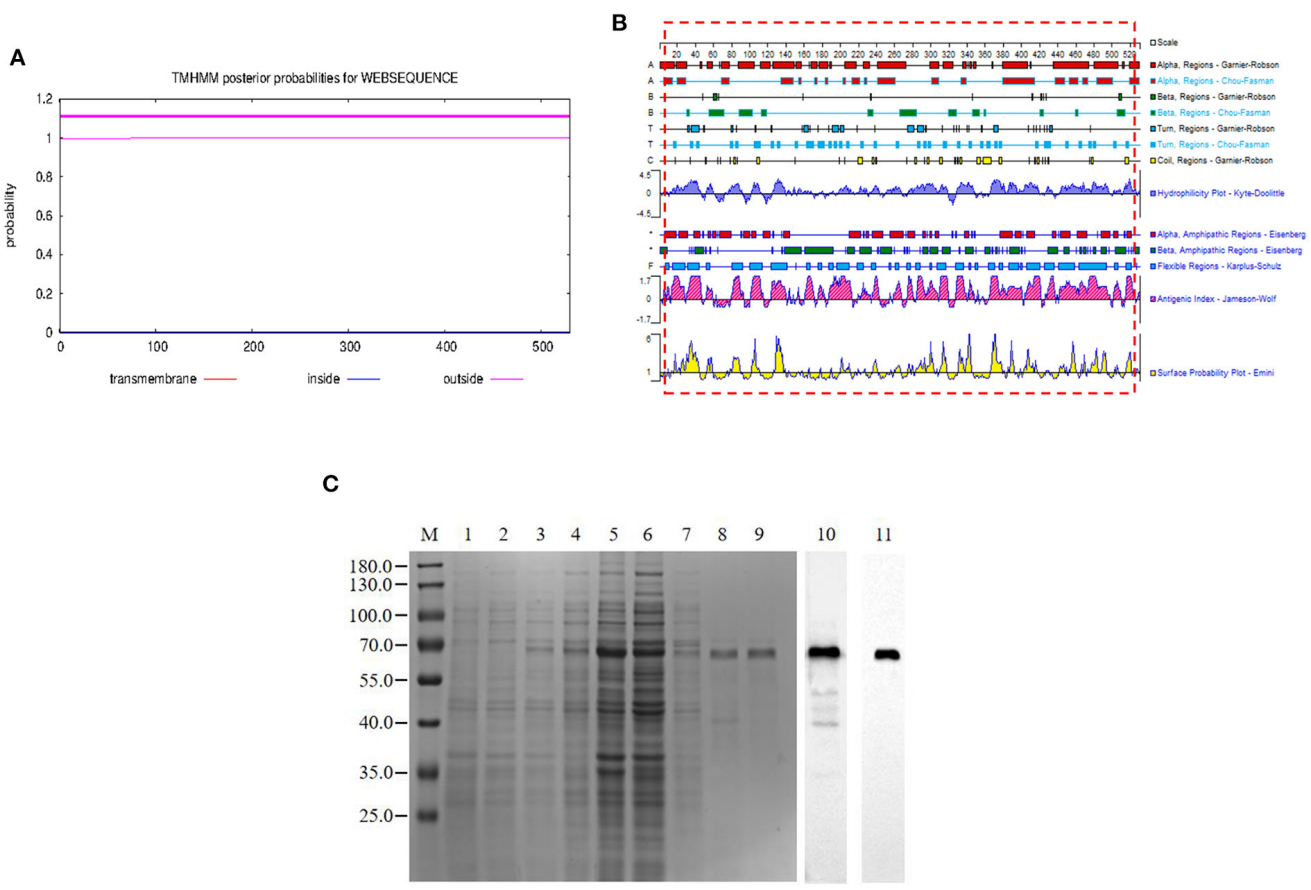
Expression and purification of the pB602L protein. **(A)** Prediction of the full-length transmembrane region of pB602L protein **(B)** Expression of pB602L protein in vector pCold I. **(C)** Protein marker, 1- transformed cells of BL21/pCold after IPTG induction for 16 h, 2- whole bacteria without induction, 3- BL21/pCold-B602L whole bacteria after IPTG induction for 16 h, 4-BL21/pCold-B602L supernatant after induction at 16°C, 5-BL21/pCold-B602L precipitation after IPTG induction for 16 h, 6-Purification of recombinant pB602L protein flow-through by Ni^+^ spin column affinity chromatography, 7-Ni^+^ spin column affinity chromatography purification of recombinant protein pB602L 40mM imidazole washing solution, 8, 9-purified recombinant pB602L protein by affinity chromatography of Ni^+^ spin column, 10-Western blot analysis of p602L protein using ASFV-positive pig serum, 11-Western blot analysis of p602L protein using anti-His. A prominent band with the expected size 65 kDa appeared after incubation.

The pB602L protein had no transmembrane region (Figure 1A). In this study, we expressed the full length of pB602L protein (Figure 1B). The 65-kDa ASFV pB602L protein was successfully expressed. It was detected in the soluble supernatant fraction by WB analysis applying anti-His and ASFV-positive pig serum (Figure 1C).

### Establishment of the pB602L-ELISA method

**Figure 2:**
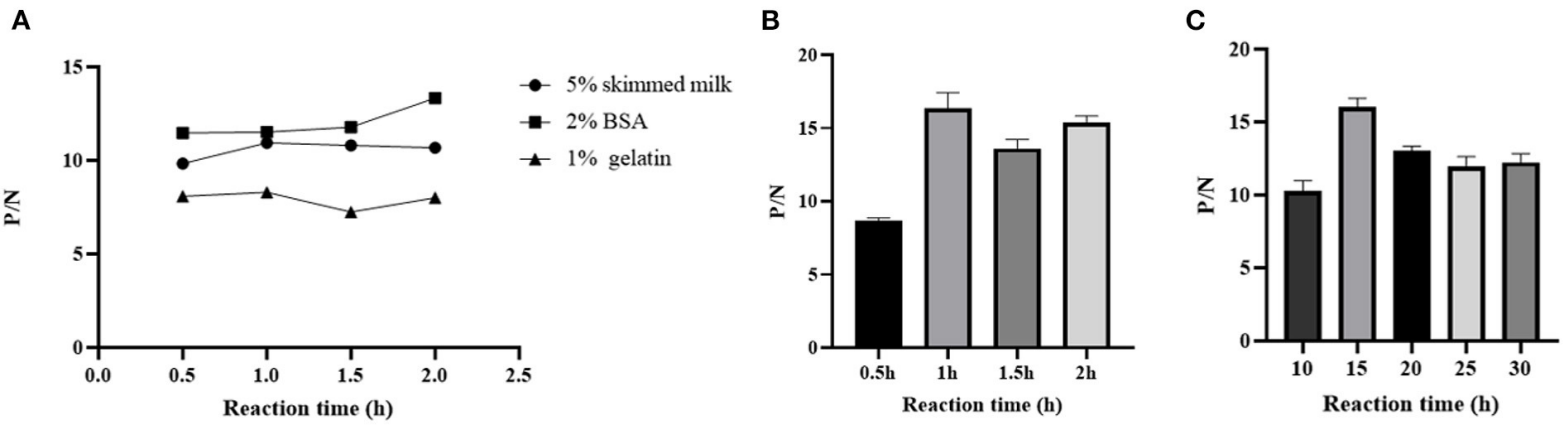
Optimization of pB602l-ELISA method conditions. **(A)** Optimization of the type of pB602L-ELISA blocking solution and working conditions. The best blocking solution was 2% BSA in PBST, and optimal blocking conditions are 2 h at 37°C. **(B)** Determination of serum action conditions. The optimal reaction time for serum was 1 h at 37 °C. **(C)** Determination of TMB solution action conditions. The optimal reaction time for TMB solution was 15 min at 37°C.

The working concentration of pB602L protein antigen and serum dilution were optimized using square titration. The experimental results show that the optimal coating concentration of antigen for the pB602L-ELISA method is 0.04 μg/mL, the optimal dilution of serum is 1:100 (Table 1). In addition, other conditions of the pB602L-ELISA method were improved. In brief, the best blocking solution is 2% BSA in PBST, and optimal blocking incubation is 2 h at 37°C (Figure 2A). The optimal reaction time for serum was 1 h at 37°C (Figure 2B). The optimal dilution of HRP-conjugated anti-pig was 1:5,000, and the optimal reaction condition was 1 h at 37°C (Table 2). Finally, the optimal reaction time for TMB solution was 15 min at 37°C (Figure 2C).

**Table 1:** P/N values at different conditions.

**Serum dilution**		**Antigen coating concentration (**μ**g/mL)**				
		**0.625**	**0.320**	**0.160**	**0.080**	**0.040**
	P	3.585	3.209	2.715	2.424	2.363
1:50	N	0.086	0.105	0.086	0.08	0.088
	P/N	41.686	30.562	31.570	30.300	26.852
	P	3.095	2.410	1.775	1.555	1.483
1:100	N	0.093	0.120	0.088	0.066	0.080
	P/N	33.279	20.083	20.170	23.561	18.538
	P	2.359	1.764	1.211	1.116	1.056
1:200	N	0.076	0.078	0.08	0.084	0.073
	P/N	31.039	22.615	15.138	13.286	14.466
	P	1.599	1.240	0.951	0.740	0.769
1:400	N	0.077	0.080	0.072	0.066	0.055
	P/N	20.766	15.500	13.208	11.212	13.982

**Table 2:** HRP-conjugated anti-pig working conditions.

**HRP anti-pig antibody dilution**		**HRP anti-pig antibody action conditions**			
		**37°C 0.5 h**	**37°C 1 h**	**37°C 1.5 h**	**37°C 2 h**
1:2,500	P	1.082	1.268	1.263	1.464
	N	0.300	0.255	0.233	0.268
	P/N	3.607	4.973	5.421	5.463
1:5,000	P	0.774	1.048	1.192	1.248
	N	0.183	0.163	0.195	0.203
	P/N	4.230	6.429	5.519	6.148
1:10,000	P	0.526	0.784	1.003	1.065
	N	0.169	0.175	0.234	0.176
	P/N	3.112	4.480	4.286	6.051

### Optimization of the cut-off value

**Figure 3:**
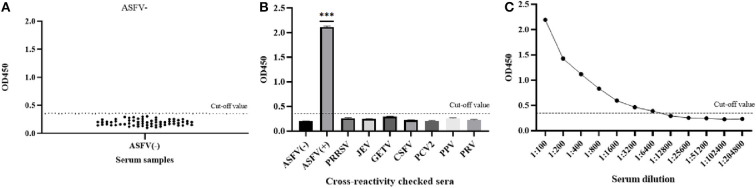
Sensitivity and specificity of the pB602L-ELISA method. **(A)** Determination of the cut-off value of pB602L-ELISA (OD450 = 0.340). Distributions of OD values determined for ASFV-negative (*n* = 64) serum samples using pB602L-ELISA. The average value (AV) of the negative sera was calculated, and the cutoff value was set at 0.340 (AV+3×SD). **(B)** The specificity test of the pB602L-ELISA method. ASFV-positive serum, ASFV-negative serum and PRRSV, JEV, GETV, CSFV, PCV2, PPV, PRV antibody-positive swine serum were measured using the pB602L-ELISA, and the OD450 values with all antisera except the ASFV-positive serum were lower than the cutoff value (0.340). **(C)** Sensitivity of the pB602L-ELISA method. Two-fold serial dilutions from 1:100 to 1:204,800 of ASFV-positive serum were tested by pB620L-ELISA method, and the highest dilution that could be detected using the cutoff value (0.340) was found to be 1:6,400, ***p < 0.001. Each dot represents a serum sample, and the dashed line indicates the cutoff value of the pB602L-ELISA.

The average OD450 value of negative sera (*n* = 64) was 0.192, and the SD was 0.049, so the cut-off value of the pB602L-ELISA method was AV + 3SD = 0.340 (Figure 3A). Therefore, samples with an OD450 value of the tested serum of ≥0.340 are considered positive. Conversely, samples with an OD450 value of <0.340 are considered negative.

### Diagnostic specificity of pB602L-ELISA

The established pB602L-ELISA method was used to test anti-PRRSV-, anti-JEV-, anti-GETV-, anti-CSFV-, anti-PCV2-, anti-PPV-, and anti-PRV-positive pig serum. Except for the ASFV-positive control, all sera were negative (OD450 < 0.340), which indicated that the established ELISA method has good specificity (Figure 3B).

### Diagnostic sensitivity of pB602L-ELISA

The ASFV-positive serum was diluted according to different dilutions. The results showed that the maximum dilution of serum that still had an OD450 value above the cutoff value of 0.340 was 1:6,400, so the sensitivity of the established pB620L-ELISA method was 1:6,400 (Figure 3C), indicating that the established indirect ELISA method is extremely sensitive.

### Repeatability of the pB602L-ELISA method

Intra-assay variation and inter-assay variation were calculated to verify the repeatability of the pB602L-ELISA method. The maximum CV of the intra-assay repeatability test was 4.27% (Table 3), and the maximum CV of the inter-assay repeatability test was 5.82% (Table 3), both <10%. These results indicate that the established ELISA method has good repeatability.

**Table 3:** The results of the repeating test.

**Sample number**	**The result of intra**			**The result of inter**		
	**Average**	**Standard**	**Coefficient**	**Average**	**Standard**	**Coefficient**
	**value**	**deviation**	**variation**	**value**	**deviation**	**variation**
1	2.062	0.088	4.27%	2.674	0.099	3.73%
2	1.809	0.012	0.68%	2.512	0.057	2.25%
3	1.754	0.045	2.56%	1.218	0.049	4.09%
4	2.160	0.044	2.05%	2.541	0.078	3.08%
5	3.064	0.047	1.55%	0.877	0.051	5.82%
6	0.805	0.017	2.05%	1.129	0.033	2.95%

### Comparison of the pB602L-ELISA method to commercial kits

The performance of the pB602L-ELISA method was compared with that of a commercial ELISA kit (ID Screen^®^ African Swine Fever Competition) for anti-ASFV detection in terms of specificity and accuracy. The results are shown in Table 4. The concordance rate of the detection results of these two methods is 97.33%.

**Table 4:** Comparison of the pB602L-ELISA method and a commercial ELISA kit.

**Detection method**		**Commercial ASFV ELISA antibody kit**		
		**Positive**	**Negative**	**Total**
pB602L-ELISA	Positive	29	3	32
	Negative	1	117	118
	Total	30	120	150
Relative sensitivity			96.66%	
Relative specificity			97.50%	
Compliance rate			97.33%	

## Discussion

ASF is a devastating and economically significant disease that can affect domestic and wild swine (7, 8). It was introduced in China in 2018, and because of its high fatality rate, disease prevention and control has become extremely important for the Chinese pig industry (4). At present, the control of the disease mainly relies on rapid diagnosis and culling of infected pigs and those in close contact with them. ASFV has caused huge economic losses to the global swine industry (21).

ASFV is complex, and its genome encodes many proteins related to immune evasion. There is currently no safe and effective vaccine available (3132). Consequently, it is very significant to develop a method that can quickly and accurately detect ASFV. At present, serological detection techniques and molecular diagnostic methods are still regarded as the main means of identifying infected animals and preventing and controlling ASF (12, 22). There are many methods for detecting viral DNA, including real-time quantitative PCR and *in situ* hybridization with nucleic acid probes (23). Although these methods have high sensitivity, they also have high requirements on experimental operation technology and the experimental environment (23, 24). Serological diagnosis based on ELISA is not only stable detection means, but it also has good specificity, high sensitivity, simple operation, and low cost, so it is appropriate for the detection of large batches of samples, and hence it is widely used in practice (22). Therefore, the OIE considered ELISA as the first serological method for the diagnosis of ASF (23). Most commercial ELISA assays have been developed based on recombinant ASFV structural proteins such as p72, p54, and p30, and these assays have been used for clinical diagnosis of porcine ASFV infection (25). At present, studies have shown that in addition to the structural proteins of ASFV, such as p72, p54, and p30, its non-structural proteins, such as pK205R and pB602L, also have good immunogenicity, so an effective ELISA method could be established based on these proteins (17, 23).

The pB602L protein of ASFV is encoded by the B602L gene, which contains a central variable region and is frequently used for subgenotyping of ASFV isolates (26). One of the proven markers to characterize this virus is the central variable region (CVR) within the B602L gene (17, 27). pB602L is an important nonstructural protein and a molecular chaperone of the major structural protein p72, which is required for the formation of the icosahedral capsid structure of ASFV (16). The B602L gene contains a central variable region, so the amino acid sequence alignment analysis of the B602L protein of different genotypes of ASFV strains was carried out. The results are shown in of the Supplementary Figures 1, 2. The B602L protein of the strain has only a few amino acid differences, and the B602L protein has a high homology in different genotype. We also performed an amino acid sequence alignment analysis of the B602L protein of the genotype II ASFV strain prevalent in China. The results are shown in of the Supplementary Figure 3. The alignment results show that the B602L protein of the known prevalent strains in China is not different. In conclusion, the overall antigenicity of ASFV B602L protein is relatively high, and there is only genotype II ASFV strain in China, so the selection of B602L protein has high sensitivity. Previous studies have shown that pB602L has good antigenicity and immunogenicity; the protein can be recognized by domestic pig and wild boar immune sera, and it may serve as a candidate antigen for the development of diagnostic methods for ASFV (28). Consistently high serological responses against pB602L were observed at time points later in infection than responses against the structural proteins in domestic pigs and bushpigs infected with a range of different viral isolates (29, 30). B Gutiérrez-Castañeda et al. show that ASFV B602L protein can be applied to antibody kinetics (17). Consistently high serological responses against pB602L was observed at time points later in infection than responses against the structural proteins in domestic pigs and bushpigs infected with a range of different viral isolates (17). So Gutiérrez-Castañeda et al. (17) suggests that an ELISA against these proteins may be of use in distinguishing animals persistently infected with virus from animals immunized with vaccines incorporating virus structural proteins (17). This suggests that an ELISA against these proteins may be of use in distinguishing animals persistently infected with virus from animals immunized with vaccines incorporating virus structural proteins. Previously, a pB602L-based ELISA assay was used to detect ASFV serum antibodies, which also distinguished pigs persistently infected with native ASFV strains from pigs immunized with subunit vaccines of structural proteins (16). Compared with eukaryotic expression systems, prokaryotic expression systems have lower production costs, simple operation, stable protein preparation, and easy purification and are suitable for large-scale production (31, 32). Therefore, in this study, a prokaryotic expression system was used to express the ASFV pB602L protein. The results revealed that the obtained B602L protein was soluble and strong antigenicity, and the B602L protein could be served as an ELISA antigen to create and improve an indirect ELISA detection assay.

The indirect ELISA antibody detection method established in this study has a sensitivity of up to 1:6,400. The established method does not cross-react with antibodies against other related swine viruses such as PRRSV, JEV, CSFV, GETV, PCV2, PPV, and PRV. The CV in not only intra-batch but also inter-batch repeatability tests were <10%, which indicated that the pB602L-ELISA method constructed in this research has favorable repeatability. The concordance rate with a commercial ELISA kit (ID Screen^®^ African Swine Fever Competition) for anti-ASFV detection was 97.33%. In conclusion, the recombinant ASFV pB602L protein prepared in this study has good antigenicity, and the established indirect ELISA antibody detection method has good sensitivity, good specificity, and high efficiency, and hence it is suitable for the on-site detection of anti-ASFV. This will provide an experimental basis for the detection of ASFV and the specific diagnosis of the disease. It has the potential to become an effective tool for evaluating the humoral immune response in pigs and the protective effects of live attenuated vaccines. Similarly, the construction of ELISA detection method is of great implication for the prevention and treatment of ASF.

## Conclusion

In this study, the pB602L-ELISA method for indirect ASFV detection was successfully established. It has good reproducibility and specificity, and production and operation are simple. This study lays the foundation for the development of a kit for large-scale detection of anti-ASFV.

## Data availability statement

The datasets presented in this study can be found in online repositories. The names of the repository/repositories and accession number(s) can be found in the article/Supplementary material.

## Author contributions

YY: formal analysis, investigation, and validation. QX: formal analysis. QS: methodology and writing–review and editing. YZ: formal analysis and investigation. YL, ZG, and JZ: investigation and validation. XM: validation. BL and YQ: validation and writing–review and editing. KL: validation. DS: methodology. ZM: conceptualization, funding acquisition, and project administration. JW: conceptualization, formal analysis, funding acquisition, investigation, methodology, and writing–original draft. All authors contributed to the article and approved the submitted version.

## Funding

The work was supported by grants from the National Key Research and Development Program of China (Nos. 2021YFD1801300 and 2021YFD1801401 awarded to JW and YQ, respectively), the National Natural Science foundation of China (No. 31941012 awarded to JW), the Project of Shanghai Science and Technology Commission (No. 20392002400 awarded to JW), and the Agricultural Science and Technology Innovation Program (CAAS-ZDRW202203 awarded to ZM). The funders had no role in the study design, data collection and analysis, the decision to publish, or preparation of the manuscript.

## Conflict of interest

The authors declare that the research was conducted in the absence of any commercial or financial relationships that could be construed as a potential conflict of interest.

## Publisher's note

All claims expressed in this article are solely those of the authors and do not necessarily represent those of their affiliated organizations, or those of the publisher, the editors and the reviewers. Any product that may be evaluated in this article, or claim that may be made by its manufacturer, is not guaranteed or endorsed by the publisher.
