# Supplementary material for: Detection of African swine fever virus antibodies in serum using a pB602L protein-based indirect ELISA
Source: Front Vet Sci. 2022 Sep 23;9:971841. doi: 10.3389/fvets.2022.971841 (PMC9540791; doi:10.3389/fvets.2022.971841)
Supplement: Supplementary file 1 [file Presentation_1.pptx]

## Slide 1
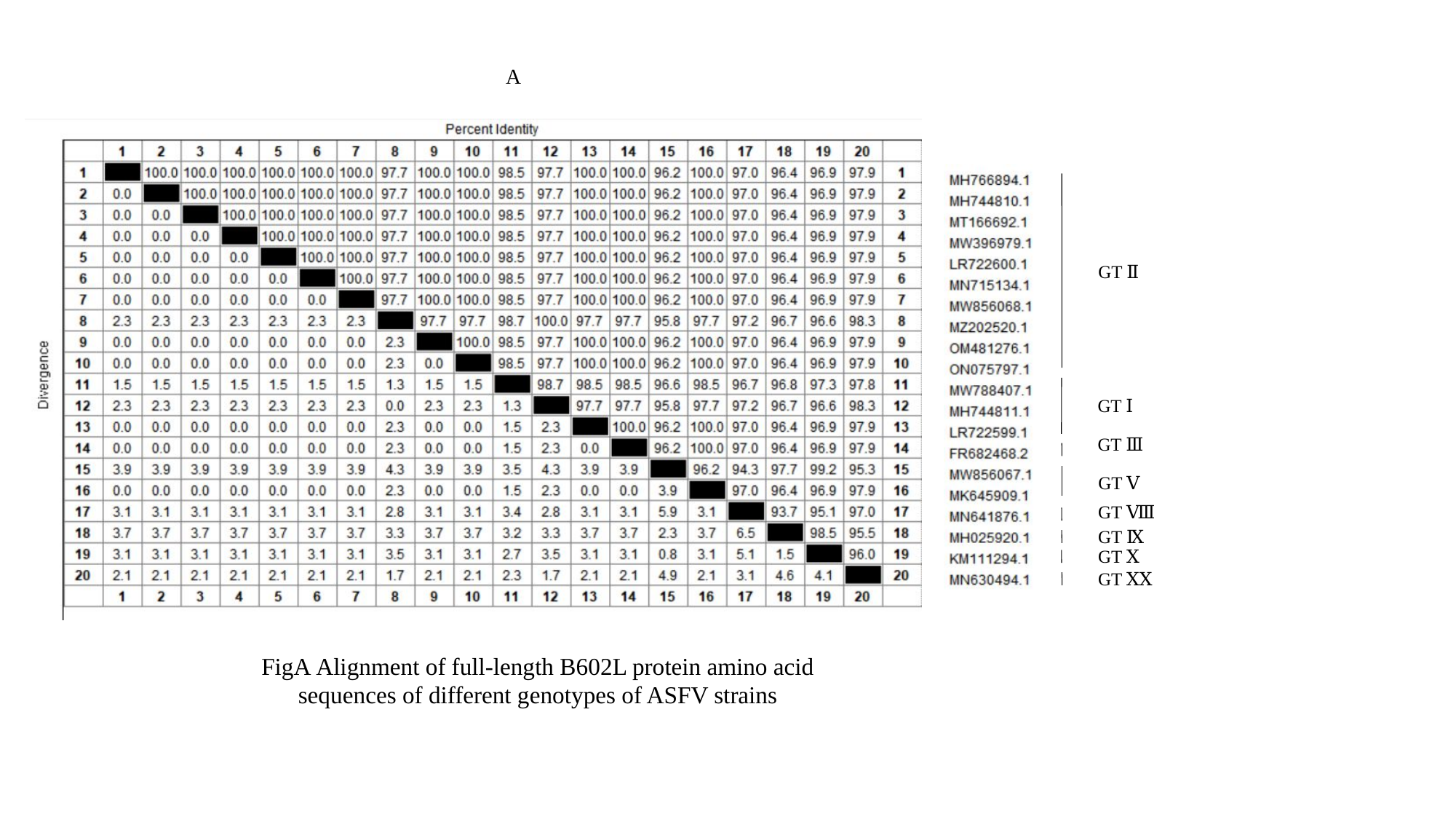

A
GT Ⅱ
GT Ⅰ
GT Ⅲ
GT Ⅴ
GT Ⅷ
GT Ⅸ
GT Ⅹ
GT ⅩⅩ
FigA Alignment of full-length B602L protein amino acid sequences of different genotypes of ASFV strains
