# Supplementary figures and images for: Detection of African swine fever virus antibodies in serum using a pB602L protein-based indirect ELISA
Source: Front Vet Sci. 2022 Sep 23;9:971841. doi: 10.3389/fvets.2022.971841 (PMC9540791; doi:10.3389/fvets.2022.971841)

## Slide 1
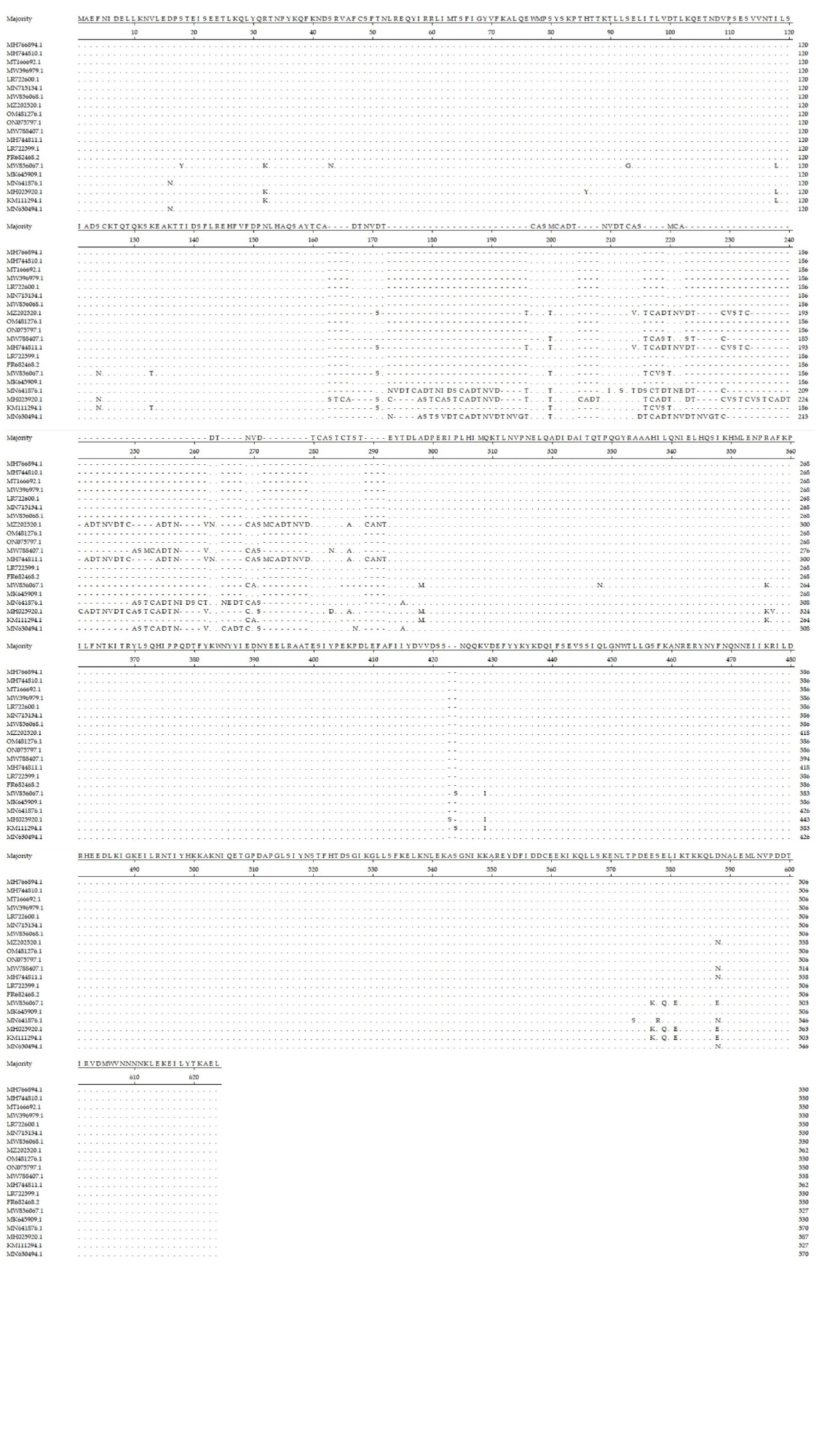

Supplement: Supplementary file 2 [file Presentation_2.pptx]

## Slide 1
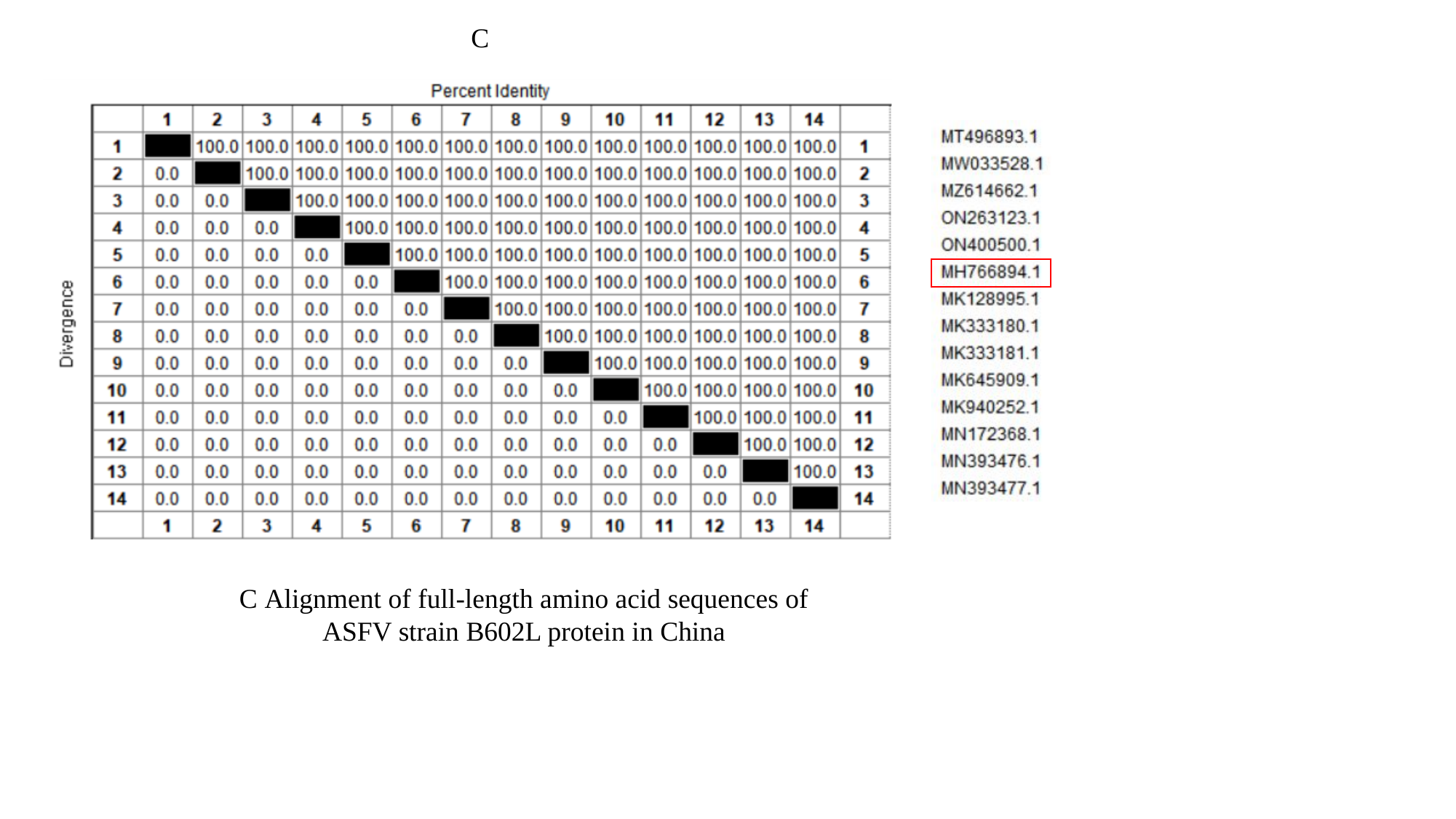

C
C Alignment of full-length amino acid sequences of ASFV strain B602L protein in China

Supplement: Supplementary file 3 [file Presentation_3.pptx]
